# Molecular characterization of an outbreak-involved *Bacillus anthracis* strain confirms the spillover of anthrax from West Africa

**DOI:** 10.1186/s40249-023-01172-2

**Published:** 2024-01-15

**Authors:** Shuchao Wang, Roland Suluku, Mohamed B. Jalloh, Ahmed F. Samba, Baogui Jiang, Yubiao Xie, Doris Harding, Mengyao Zhang, Foday Sahr, Mahmud E. Sesay, James S. Squire, Mohamed A. Vandi, Moinina N. Kallon, Shoufeng Zhang, Rongliang Hu, Yuee Zhao, Zhiqiang Mi

**Affiliations:** 1 Changchun Veterinary Research Institute, Chinese Academy of Agricultural Sciences, Changchun, China; 2 Department of Animal Sciences, School of Agriculture and Food Sciences, Njala University, Njala, Sierra Leone; 3 Department of Microbiology, College of Medicine and Allied Health Sciences, University of Sierra Leone, Freetown, Sierra Leone; 4 Ministry of Agriculture and Food Sciences, Freetown, Sierra Leone; 5 Beijing Institute of Microbiology and Epidemiology, 20 East Street, Fengtai District, Beijing, China; 6 Ministry of Health and Sanitation, Freetown, Sierra Leone; 7 Beijing Institute of Biotechnology, Beijing, China

**Keywords:** Molecular identification, Anthrax, *Bacillus anthracis*, Sierra Leone, Phylogeny, Single nucleotide polymorphism

## Abstract

**Background:**

Anthrax, a zoonotic disease caused by the spore-forming bacterium *Bacillus anthracis*, remains a major global public health concern, especially in countries with limited resources. Sierra Leone, a West African country historically plagued by anthrax, has almost been out of report on this disease in recent decades. In this study, we described a large-scale anthrax outbreak affecting both animals and humans and attempted to characterize the pathogen using molecular techniques.

**Methods:**

The causative agent of the animal outbreak in Port Loko District, Sierra Leone, between March and May 2022 was identified using the nanopore sequencing technique. A nationwide active surveillance was implemented from May 2022 to June 2023 to monitor the occurrence of anthrax-specific symptoms in humans. Suspected cases were subsequently verified using quantitative polymerase chain reaction. Full-genome sequencing was accomplished by combining long-read and short-read sequencing methods. Subsequent phylogenetic analysis was performed based on the full-chromosome single nucleotide polymorphisms.

**Results:**

The outbreak in Port Loko District, Sierra Leone, led to the death of 233 animals between March 26th and May 16th, 2022. We ruled out the initial suspicion of *Anaplasma* species and successfully identified *B. anthracis* as the causative agent of the outbreak. As a result of the government's prompt response, out of the 49 suspected human cases identified during the one-year active surveillance, only 6 human cases tested positive, all within the first month after the official declaration of the outbreak. The phylogenetic analysis indicated that the BaSL2022 isolate responsible for the outbreak was positioned in the A.Br.153 clade within the TransEuroAsian group of *B. anthracis*.

**Conclusions:**

We successfully identified a large-scale anthrax outbreak in Sierra Leone. The causative isolate of *B. anthracis*, BaSL2022, phylogenetically bridged other lineages in A.Br.153 clade and neighboring genetic groups, A.Br.144 and A.Br.148, eventually confirming the spillover of anthrax from West Africa. Given the wide dissemination of *B. anthracis* spores, it is highly advisable to effectively monitor the potential reoccurrence of anthrax outbreaks and to launch campaigns to improve public awareness regarding anthrax in Sierra Leone.

**Graphical Abstract:**

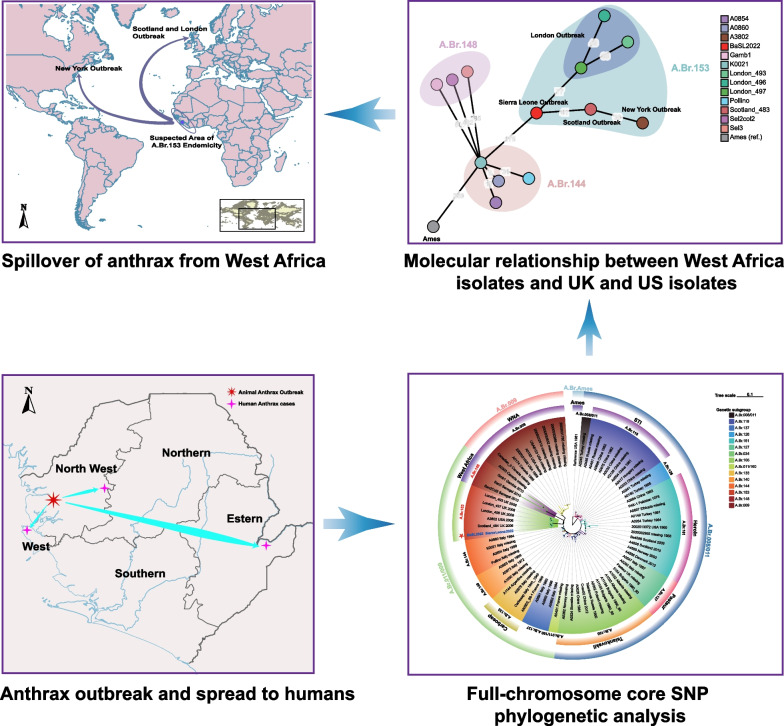

**Supplementary Information:**

The online version contains supplementary material available at 10.1186/s40249-023-01172-2.

## Background

Anthrax is a zoonotic disease with a significant historical background. Despite being a long-standing issue, it remains a prominent global public health concern, particularly in resource-limited regions [1]. The disease, affecting both humans and animals, is caused by *Bacillus anthracis*, a gram-positive, spore-forming bacterium. Infection with *B. anthracis* typically results in cutaneous, gastrointestinal, inhalational, or injectional anthrax. Cutaneous anthrax is the prevailing form in humans, accounting for over 95% of reported human cases [1]. This form of anthrax typically has a comparatively lower mortality rate, whereas gastrointestinal and inhalational forms of anthrax, although less prevalent, are characterized by a significantly higher lethality rate and are considered the primary cause of most anthrax-related fatalities in humans [2, 3]. Unlike humans, both wild herbivores and livestock predominantly encounter acute and lethal consequences as a result of gastrointestinal exposure to spores while grazing [1, 4]. Livestock anthrax frequently serves as a source of secondary human infections, mainly due to the handling and consumption of animal products that are inadvertently contaminated [5, 6]. According to available data, approximately 1.83 billion individuals were considered to be living in areas at risk of anthrax infection [3]. According to estimates, approximately 1.1 billion livestock are susceptible to the disease, mainly in rural rainfed systems, with a particular emphasis on sub-Saharan Africa [1].

Africa is one of the most threatened continents, facing the challenge of anthrax outbreaks. These outbreaks have been extensively documented in multiple countries, affecting wildlife, domestic animals, and humans alike [1, 5–8]. However, it is believed that the actual number of cases is considerably underestimated due to inadequate diagnostic capabilities [8, 9]. Based on modeling techniques, previous studies have identified several regions in eastern, central, and southern Africa, as well as an east‒west corridor spanning from Ethiopia to Sierra Leone in the Sahel region, as having favorable conditions for the spread of *B. anthracis* [8, 10]. However, thus far, only Botswana, Zambia, Uganda, Tanzania, Namibia, South Africa, Nigeria, Cameroon, Chad, Ghana, and Ethiopia have documented verified instances of anthrax through the utilization of molecular and/or bacteriological techniques [10–12]. In Sierra Leone, sporadic instances of suspected anthrax can be identified in the annual reports of the Ministry of Agriculture between 1947 and 1994. The latest recorded outbreak data date back to 1994, during which 8 individuals lost their lives as a result of consuming deceased goat meat sourced from Guinea (Personal interview). Recently, there have been four reported cases of suspected cutaneous anthrax in human individuals within the vicinity of Kamakwie, Sierra Leone [13]. Regrettably, none of these records have been supported by dependable molecular or validated bacteriological methods. The absence of molecular verification significantly impedes the utility of these outbreak records as a reliable genetic resource for disease control.

*B. anthracis* is characterized by a relatively monomorphic nature, exhibiting a highly conserved genome composition. This implies that the genetic relatedness between descendant strains can be readily ascertained through the utilization of genomic molecular techniques, such as multilocus variable-number-of-tandem-repeats analysis (MLVA) and canonical single nucleotide polymorphisms (SNPs) [14, 15]. According to the analyses conducted using MLVA and the 12 canonical SNPs, *B. anthracis* exhibited a phylogenetic division into three primary branches, namely, A, B, and C. These branches were further classified into several sublineages, each characterized by distinct geographical distributions [14, 16]. With the emergence of cost-effective whole-genome sequencing methods, the field of phylogenetic reconstruction has witnessed a significant increase in the utilization of genome-wide SNPs as well as core-genome-based multilocus sequence typing (cgMLST) [16–19]. These methods offer improved strain clustering resolution, rendering them a potent tool for tracing outbreaks and modeling anthrax transmission [20–22]. Among the three primary phylogenetic branches of *B. anthracis*, isolates belonging to the B and C branches are exceedingly uncommon and geographically limited, often found only in specific locations or archival sources. In contrast, the A branch of *B. anthracis* exhibits a global distribution and accounts for more than 90% of outbreaks [14]. Within the A branch, the majority of the sublineages demonstrate a distinct geographical distribution. However, the TransEuroasian (TEA) sublineage has achieved significant global dissemination, possibly due to a rapid clonal radiation event within a short timeframe. This is evident from the remarkably short phylogenetic branches [21, 22]. In the West African region, there exists a limited number of publicly accessible complete genomes of *B. anthracis*, which were all isolated in Senegal and Gambia [23]. All these isolates belong to the TEA sublineage and exclusively form a specific subclade that has been recently designated as A.Br.148, according to the nomenclature of the full-chromosome SNP phylogeny system [19, 22]. Of note, despite the constraints of public genomic resources, West Africa bears a significant burden of anthrax caused by *B. anthracis* lineages that are primarily prevalent in this specific region [6, 9, 24]. A notable illustration is the exclusive presence of the specific lineage A.Br.148 in West Africa. This *B. anthracis* lineage, coupled with a distinct lineage found in Cameroon, Chad, Mali, and Nigeria that lacks spore-surface-associated anthrose [12, 25, 26], as well as the nontypical *B. anthracis* strains isolated from wild apes in Côte d'Ivoire and Cameron [27, 28], provides a glimpse into the complex nature of anthrax epidemiology in West Africa.

In this study, we provide a detailed account of an anthrax outbreak in Sierra Leone that exhibited an unprecedented magnitude. This outbreak led to the unfortunate demise of more than 200 animals and caused infections in six individuals. The analysis of the full-genome sequence indicated that the strain responsible for the outbreak was distinct from all existing West African lineages but may be associated with the anthrax outbreaks in London, Scotland, and New York between 2006 and 2008.

## Methods

### Sample collection during the outbreak and the study setting of the post-outbreak active surveillance

**Fig. 1:**
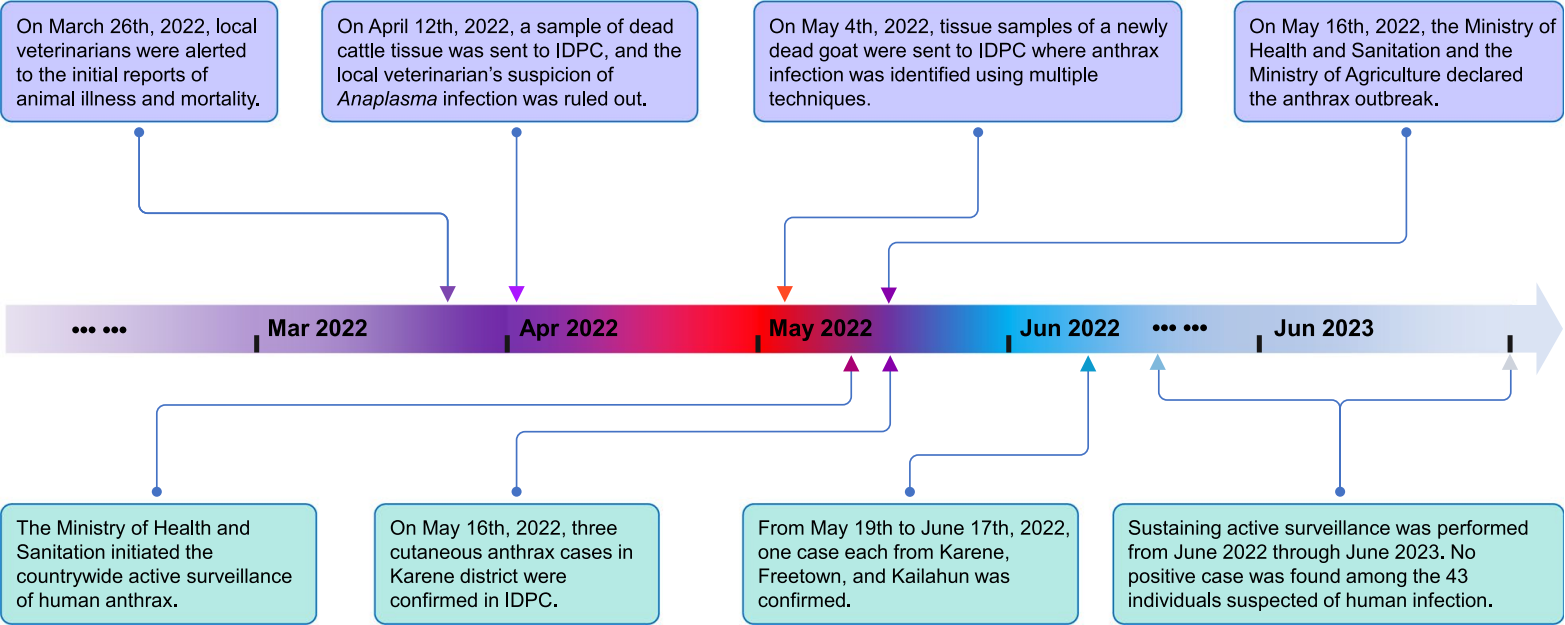
The identification and official response to the anthrax outbreak in Port Loko District of Sierra Leone. The upper half of the diagram shows time points related to animal outbreaks in lilac, while the lower half depicts time points associated with human cases in cyan. *IDPC* The tropical Infectious Diseases Prevention & Control Center of Sierra Leone

On March 26th, 2022, herbivorous livestock in Thinka Barreh village (8°48′32″N, 12°55′40″W) of the Bakeloko Chiefdom, located in the Port Loko District of Sierra Leone, were reported to be afflicted by an unidentified disease (Fig. 1). On April 11th, samples of tissue from the spleen and liver of a deceased bovine, along with blood samples from eight live bovines, were collected from the Affina Jalloh farm and submitted to the Tropical Infectious Diseases Prevention and Control Center of Sierra Leone (IDPC) for verification of the initial suspicion of *Anaplasma* infection, as reported by local veterinarian. On May 4th, a comprehensive collection of clinical samples was conducted, comprising 40 blood samples from live cattle and 6 blood samples from live sheep, as well as tissue samples from the heart, liver, spleen, lung, and kidney of a newly deceased sheep. These samples were collected from two neighboring farms, Chernaor Bah and Amadu Wurie Bah, where the grazing areas overlapped with the Affina Jalloh farm. After collection, the samples were promptly dispatched to the IDPC in a cold chain for the molecular identification of the causative agent.

As an effort to effectively control the outbreak, prompt implementation of active surveillance was carried out following its identification. Sierra Leone is a country with a population of approximately 7.54 million inhabitants. It is administratively divided into five Regions and sixteen Districts, which are further subdivided into Chiefdoms, followed by villages and communities. In the context of active surveillance, a comprehensive approach was employed to encompass all villages and communities across the entire nation. The active surveillance commenced on May 16th, 2022, and was concluded in June 2023, covering both the rainy and dry seasons.

### Rapid identification of the causative agent responsible for the outbreak

All experimental procedures were conducted within a designated A2-type biological safety cabinet situated in a biosafety level 3 (BSL-3) facility. The liver sample underwent two rounds of rinsing with ethanol (70%) and was subsequently divided into smaller sections. The materials underwent a washing process using 1 ml of distilled water, and 200 μl of the supernatant was utilized for the extraction of DNA/RNA using the TGuid S32 magnetic DNA/RNA isolation kit (Tiangen, Beijing, China) following the manufacturer's instructions. The nucleic acid was sterilized at 95 °C for 20 min. The concentration of the nucleic acid was determined using a Qubit 4 fluorometer (Invitrogen, Carlsbad, USA). A nanopore sequencing library was prepared by utilizing the DNA samples and the Rapid Barcoding and Sequencing kits provided by Oxford Nanopore Technologies (Oxford, UK). The library was subsequently subjected to sequencing on a MinION Mk1B device using an R9.4.1 flow cell (Oxford Nanopore Technologies, Oxford, UK). During the one-hour sequencing process, the acquisition of raw data, base calling, and demultiplexing were carried out in real time using MinKNOW software associated with the device, utilizing a "superaccuracy" model. A swift annotation was then performed using the software Centrifuge (release 1.0.3) with the "Bacteria, Archaea, Viruses, Human (compressed)" database [29].

### Confirmation of *B. anthracis* through quantitative PCR (qPCR) and cultivation

Tissue samples were processed according to the aforementioned procedure, with a minor adjustment. A homogenization process was performed on each tissue sample, where 200 mg of the sample was mixed with 1 ml of normal saline solution. A volume of 200 μl of the supernatant obtained from the tissue homogenate or blood sample was used for DNA extraction. The analysis of *B. anthracis* DNA was conducted through qPCR to examine the presence of chromosomal marker *dhp61* (BA_5345) as well as plasmid markers *pagA* (pXO1) and *capC* (pXO2) using the primers as previously described [30, 31].

For cultivation, the liver tissue sample was subjected to two rounds of rinsing with ethanol (70%) and distilled water and subsequently partitioned into two distinct sections. The cutting plane was applied onto the nutrient agar, which was incubated at a temperature of 37 °C for 24 h. Colonies were subsequently purified using the streaking method. One colony was selected to prepare a slide for Gram staining.

### Procedure of the active surveillance

Post-outbreak active surveillance of human anthrax was carried out in compliance with the directive of the Ministry of Health and Sanitation. Community health workers were given instructions to identify cases displaying typical symptoms indicative of suspected anthrax. These symptoms encompass the manifestation of a painless or itchy papule accompanied by excessive swelling, which subsequently progresses into a vesicular morphology, ruptures, and eventually forms an ulcer and black eschar [4]. If individuals exhibiting such symptoms had been in close proximity to, or had come into contact with, animal meat sourced from Port Loko District within a two-week period prior to the manifestation of symptoms, a swab specimen and a whole blood sample were collected. These samples were expeditiously transferred to the IDPC in a cold-chain system within 24 h. In the BSL-3 laboratory, the samples were subjected to analysis using qPCR and cultivation techniques, as previously stated. Samples that tested positive for both chromosome and plasmid markers were classified as confirmed cases.

### Full-genome sequencing

Cells from the culture were harvested and digested with lysosome at 20 mg/ml and RNase A at 2 mg/ml for 1 h, followed by genomic DNA extraction using the TGuide Bacteria DNA Kit (Tiangen, Beijing, China) following the manufacturer’s instructions. Sterilization of the resulting DNA was performed using 0.22-μm filters, which was further confirmed by culturing on nutrient agar. Eligible DNA with no positive colonies on the plate within 72 h was subsequently quantified using the Qubit 4 fluorometer and transferred out of the BSL-3 laboratory [17]. Full-genome sequencing was conducted by a combination of short-read sequencing and long-read sequencing techniques [32, 33]. For long-read sequencing, a nanopore sequencing library was prepared employing the Ligation Sequencing Kit and the Native Barcoding Kit (Oxford Nanopore Technologies, Oxford, UK). The library was sequenced on a MinION Mk1B device for 24 h using an R9.4.1 flow cell (Oxford Nanopore Technologies, Oxford, UK) following the manufacturer's manuals. Base calling, demultiplexing, and de novo assembly of the long-read sequencing data were performed using the corresponding pipelines and tools in EPI2ME application v5.1.1 (Oxford Nanopore Technologies, Oxford, UK) [17].

Short-read sequencing was performed on an Illumina MiSeq platform using the Nextera XT DNA Library Preparation Kit (Illumina, San Diego, USA) and the MiSeq Reagent Kit v3 (2 × 300 bp) chemistry (Illumina, San Diego, USA). High-quality paired-end reads were subjected to de novo assembly into contigs in SPAades 3.11.1 [34] prior to further refining by SNP and indel correction using SAMtools 1.7 and Pilon 1.22 [35, 36]. The assembly and polishing of the combined long reads and short reads data were ultimately conducted using MicroPIPE v0.9 [37]. The primary data generated in this study were deposited in the NCBI Sequence Read Archive (SRA) repository under the BioProject number PRJNA875505.

### Full-chromosome SNP calling and phylogenetic construction

To determine the phylogenetic position of the *B. anthracis* strain isolated in this outbreak, we retrieved 236 representative full genome sequences that were used in the initial global phylogenetic reconstruction [22] and the most recent molecular typing practice based on full-chromosome SNPs of *B. anthracis* [32]. The Parsnp tool from the Harvest Suite [38] was then used for the core chromosome multiple alignment with the ‘Ames Ancestor’ (NC_007530.2) stain serving as the reference chromosome. Chromosome-wide core SNPs were called and exported as concatenated SNP sequences. HarvestTools v1.2 from the same suite was employed to generate a variant calling file (vcf) listing all SNP-positions [32, 39]. Adjacent SNP positions, as well as sites with unknown nucleotides (N), were manually removed in the vcf for the purpose of enhancing data quality [39]. According to this curated vcf, the concatenated SNP sequences were renewed using the HarvestTool v1.2. These new SNP sequences were employed to infer the phylogenetic trees with the maximum likelihood model in RAxML v8.2.12 [40]. Phylogenetic trees were annotated using the online iTol platform [41]. A minimum spanning tree was calculated in Grapetree [42] with the curated vcf (binary format) as an input [42].

## Results

### Identification and confirmation of the anthrax outbreak in Sierra Leone

**Fig. 2:**
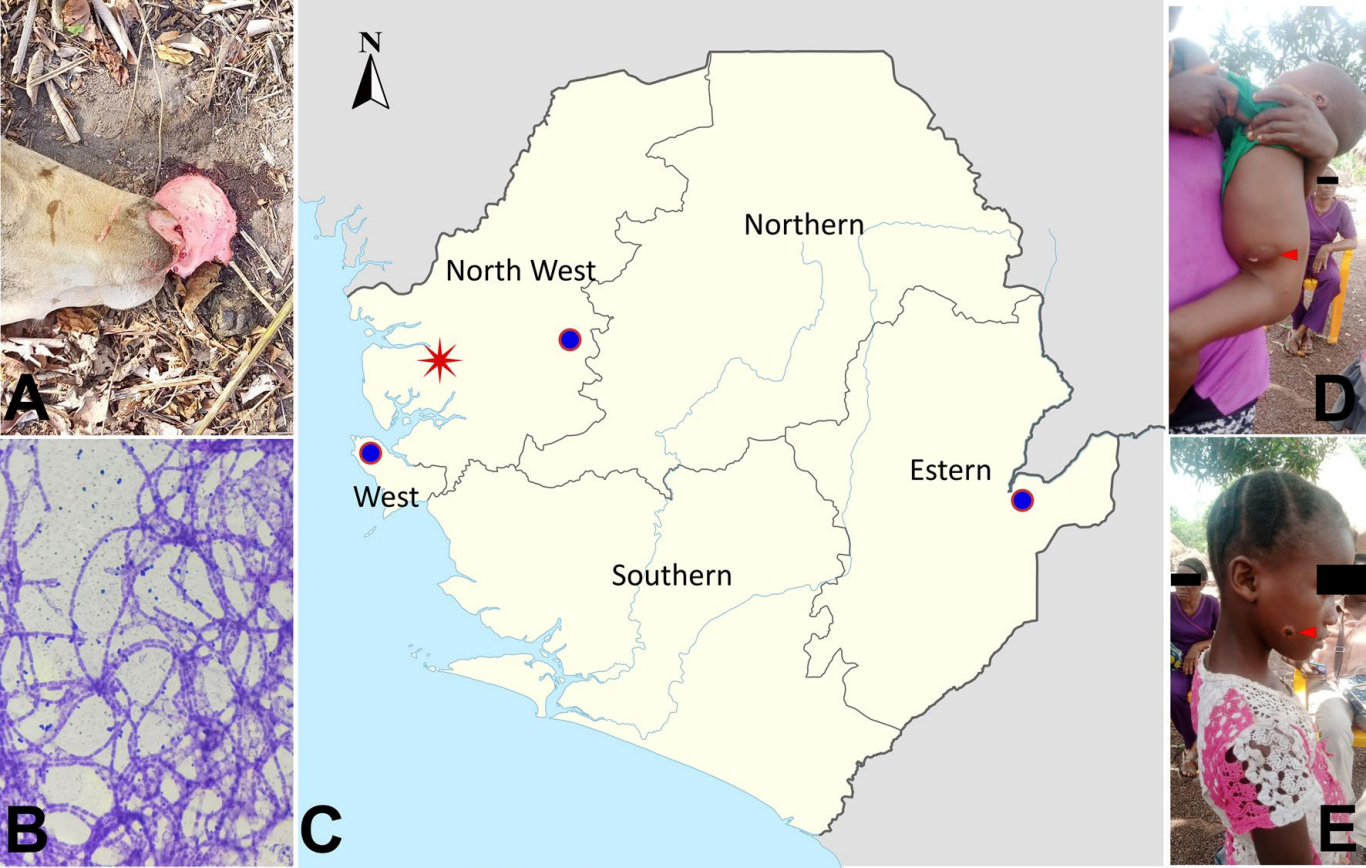
Anthrax broke out in animals in Port Loko District and spread across the country to humans. **A** The head of a diseased cow at site of death with postmortem hemorrhage from the oral and nasal cavities. **B** Gram staining of a culture taken from the liver of a sheep deceased during the outbreak (magnification 400 ×). **C** The geographical locations of the outbreak and cases. The red star marks the location of the initial animal outbreaks. The circles represent the locations of the Freetown case (left), the Karene cases (middle), and the Kailahun case (right). **D**, **E** Cases of human anthrax infections from Karene (red arrow)

The salient aspects of the outbreak are illustrated in Fig. 1. The diseased animals exhibited a range of symptoms including weakness, depression, shivering, mortality, and postmortem hemorrhage from the oral and nasal cavities (Fig. 2A). Local veterinarians hypothesized that the outbreak could potentially be attributed to a severe infection caused by *Anaplasma* species, given the observation of leukemia cases in cattle on the same farm. In the BSL-3 laboratory, a PCR-based screening was conducted on the bovine samples collected on April 11th, 2022 to identify the presence of the *Anaplasma* species [43]. The screening revealed that all samples yielded negative results, thereby refuting the initial hypothesis.

On May 3rd, the Department of Animal Sciences of Njala University led a team to the outbreak area in attempt to identify the etiology of the ongoing outbreak, as the disease's rapid dissemination resulted in substantial livestock mortality, leading to significant economic losses for farmers. Following the collection of samples, we promptly conducted an identification of the potent causative agent utilizing a random long-read sequencing technique, which has been demonstrated to be efficacious in emergency preparedness [17]. The results revealed a significant presence of anthrax infection in the deceased sheep, as the majority of the sequencing data from this sample were identified as originating from *B. anthracis* or closely related *Bacillus cereus* and *Bacillus thuringiensis*, with the exception of host sequences (Appendix, Table S1). Given the inherent error-prone nature of long-read sequencing, we conducted additional validation of the results by employing the qPCR method to assess the presence of the chromosomal marker *dhp61* (BA_5345), as well as the plasmid markers *pagA* (pXO1) and *capC* (pXO2) of *B. anthracis* in the samples. It was not surprising that the DNA samples collected from the spleen, liver, kidney, and lung all yielded strong positive results, as evidenced by cycle threshold (Ct) values ranging from 14 to 22. In contrast, all samples obtained from live animals tested negative for *B. anthracis* genes. These observations clearly indicated that when considering the Ct value of the positive control (which was 31), there was a very high bacterial load in the tissue sample of the sheep that died in the outbreak. In addition, a qPCR analysis was conducted on the bovine samples used for *Anaplasma* screening, yielding comparable outcomes. The analysis of the tissue samples exhibited a positive curve with low Ct values (ranging from 15–20), indicating the significant presence of *B. anthracis*. Conversely, the blood samples obtained from live animals tested negative for *B. anthracis*. Additional isolation and purification of *B. anthracis* from the liver and spleen tissues of the deceased sheep were performed through culturing techniques. Gram staining of the colony revealed a unique morphology characterized by elongated and rod-shaped structures, displaying a gram-positive appearance (Fig. 2B). The aforementioned findings provided compelling evidence supporting the assertion that the outbreak was indeed a result of a *B. anthracis* infection.

As of May 9th, the Affina Jalloh farm reported that 66.7% (14 out of 21) of the cattle, 100% (5 out of 5) of the sheep, and 100% (4 out of 4) of the goats had been affected by the disease. Similarly, the Chernor Bah farm reported that 31 out of 85 cattle (36.5%), 18 out of 20 sheep (90%), and 16 out of 16 goats (100%) had also perished due to the disease. These numbers signified a substantial outbreak. In contrast, in an effort to mitigate the economic losses, the farmers sold the meat of the animals that succumbed to the outbreak in the market. This action led to the extensive spread of the threat, which originated from the Port Loko District and had implications for the entire nation, affecting both animal and human populations. Consequently, the findings were promptly communicated to the Ministry of Agriculture.

### The official response and active surveillance

On May 16th, 2022, the Ministry of Agriculture and the Ministry of Health and Sanitation collaboratively announced the official declaration of an anthrax outbreak to effectively mitigate and reduce the risk of transmission associated with the emergency. Subsequently, a comprehensive set of control measures was implemented, encompassing stringent regulations that govern the production, processing, and marketing of livestock and livestock products. As of the declaration of the outbreak, a cumulative number of 233 livestock (91 cattle, 53 goats, and 79 sheep) had been reported as deceased in the impacted region, with a significant portion of the meat having entered the market. The Ministry of Health and Sanitation expeditiously implemented an enhanced surveillance program for anthrax within the local communities. On May 16th, a total of four samples (consisting of three swabs and one blood sample) were collected from seven suspected human cases in a village located in the Karene District (Fig. 2C) and subsequently submitted to the IDPC, where all three swabs tested positive for *B. anthracis* by qPCR. All three confirmed cases exhibited a distinctive symptom of cutaneous anthrax (Fig. 2D, E) and had been in close proximity to deceased livestock within two weeks. Between May 19th and June 17th, an additional three cases of cutaneous anthrax were confirmed in the IDPC. These cases were reported in the Districts of Karene, Freetown, and Kailahun, as shown in Fig. 2C. All three cases had a history of contacting animal meat within a week prior to the onset of symptoms. Since June 2022, the Ministry of Health and Sanitation has implemented an ongoing active surveillance program to monitor the occurrence of anthrax and other diseases characterized by skin lesions. Over a twelve-month duration that included both a rainy season and a dry season, a total of 43 samples obtained from individuals with suspected cases were subjected to testing for anthrax using both qPCR and cultivation methods [4, 11]. The results of these tests revealed that none of the 43 samples exhibited any traces of anthrax.

### The phylogenetic placement of the *B. anthracis* strain responsible for the outbreak

**Fig. 3:**
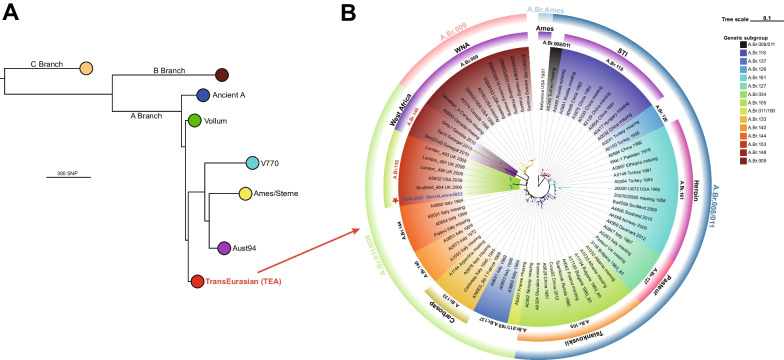
BaSL2022 was placed into A.Br.153 clade within the TranEuroAsian genetic group based on core-genome SNP analysis. **A** Global distribution of BaSL2022 shown in a maximum likelihood tree. The phylogenetic tree was constructed using core-chromosome SNPs of the representative genomes described previously [22, 32, 39]. Lineages are categorized and labeled into traditional groups. The TEA group, including BaSL2022, is highlighted in red. **B** The specific position of BaSL2022 within the TEA group. The phylogenetic tree was constructed using the maximum likelihood model. It included 72 representative genomes from the TEA group, as well as BaSL2022, with the reference strain being Ames and serving as the outgroup. The tree was drawn to scale, accurately representing the length of the branches. Nodes with a bootstrapping value above 90 are indicated by a black circle, while those between 70 and 90 are indicated by a gray circle. The lineages are displayed with the isolate's name, country of isolation, and year of isolation. The color blocks on the leaves represent the genetic subgroups of the TEA group, and the nicknames of these subgroups are also displayed in colored blocks outside the lineages. The outer layer displays the canonical SNP groups of isolates within the TEA group using circular blocks. The BaSL2022 is marked with star and shown in blue

From the tissue samples obtained from deceased cattle and sheep, we successfully isolated and purified two strains of *B. anthracis*. The integration of long-read sequencing data and short-read sequencing data derived from the genomic DNA sample [37] resulted in the identification of three expected contigs, encompassing the complete genome as well as two virulent plasmids, pXO1 and pXO2. The average sequencing depth achieved in this study was 124-fold. The application of short-read sequencing data for polishing purposes effectively mitigated the indel-introducing effect commonly associated with the long-read sequencing technique [37], thereby exhibiting a high level of reliability [17, 32]. The full-chromosome SNP sequences were identical between the two isolates. Consequently, considering the shared origin of these samples from a single outbreak, we categorized them as a unified strain, denoted as BaSL2022 (*Bacillus* Sierra Leone 2022). The construction of the global dendrogram of the *B. anthracis* phylogeny involving the analysis of 236 representative genomes and BaSL2022 was conducted using the maximum likelihood method [14], which positioned BaSL2022 within the A.Br.008/009 (TEA) sublineage (Fig. 3A) of the A branch. The precise phylogenetic placement of BaSL2022 within the TEA group is illustrated in Fig. 3B, which was generated using 72 representative genomes from the TEA lineage. BaSL2022 was classified within the canonical SNP group A.Br.011/009, more specifically falling under the A.Br.153 subgroup, which was strongly supported by bootstrapping analysis.

**Fig.4:**
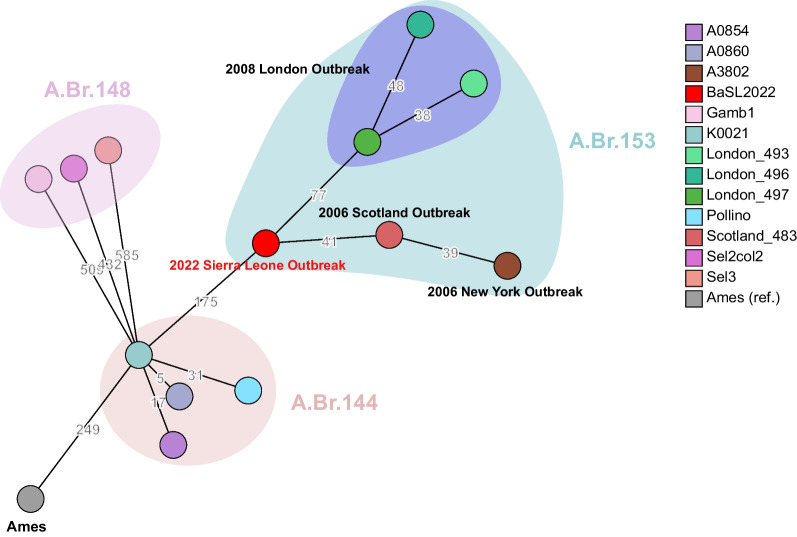
BaSL2022 phylogenetically bridged A.Br.153 subclade and the neighboring lineages. Minimum spanning tree was calculated using the core-chromosome SNP profile by Grapetree [42]. Numerical SNP distances between the chromosomes of isolates in A.Br.153, A.Br.148, and A.Br.144 are depicted on the branches. The BaSL2022 is displayed in bright red. Outbreaks associated with the isolates in A.Br.153 are also presented

Based on the core-chromosome SNPs of the isolates belonging to A.Br.153, A.Br.148, and A.Br.144, a minimum spanning tree was calculated and showed that BaSL2022 played a crucial role in connecting the evolutionary path from A.Br.144 (Fig. 4). The closest SNP distance observed between the A.Br.153 subclade and previously identified subclades was 175, specifically, between isolate BaSL2022 and Pollino (A.Br.144). The A.Br.153 subclade is exclusively characterized by lineages that are linked to the outbreaks in London, Scotland, New York, and Port Loko (Sierra Leone). Among the lineages implicated in these four outbreaks, the lineages associated with the London outbreak formed a distinct cluster and exhibited a closest genetic distance of 77 SNPs to BaSL2022. The Scotland isolate exhibited a direct genetic connection to BaSL2022, with a difference of 41 SNPs (Fig. 4).

## Discussion

Despite the extensive documentation of anthrax in West Africa, there exists a notable dearth of comprehensive data, particularly pertaining to the diagnosis and genetic sequences of *B. anthracis*, within this region. In the present investigation, we successfully identified an anthrax outbreak impacting both animals and humans in Sierra Leone through the utilization of sequencing and molecular techniques. This advancement signifies a noteworthy achievement in the realm of anthrax prevention and control in Sierra Leone.

In this anthrax outbreak, the mortality of 233 animals in Sierra Leone represents the highest number ever reported in Sierra Leone. Although laboratory confirmation was not conducted for the majority of the animal cases, the confirmation of anthrax infection in the sequentially deceased bovine and sheep, which were determined to be caused by the same strain, provides strong evidence to suggest that the suspected animal cases succumbed to anthrax exposure. From 1947 to 1994, Sierra Leone witnessed a succession of outbreaks that impacted both livestock and humans, as documented in the annual reports of the Ministry of Agriculture, with a particular focus on the Kamakwie area situated in the Northern Region. However, all these instances were diagnosed based solely on symptoms and, at most, with the assistance of microscopy [44]. This situation arose as a result of inadequate laboratory capacities and facilities, which were further compromised during the onset of the civil war in 1991. Such a predicament could potentially provide an explanation for the absence of any officially recorded instances of anthrax cases in the country since 1994. The scarcity of publicly accessible data on the disease further posed a potential contributing factor to the underdiagnosis and misdiagnosis of the disease [7, 44, 45]. The misdiagnosis also occurred at the beginning of the investigation of this anthrax outbreak, which notably hampers the timeliness of the implementation of intervention measures. In view of this, the identification of a widespread outbreak by employing nanopore sequencing to examine clinical samples of an unidentified ailment [17], as demonstrated in this study where we rapidly determined the causative agent without any preconceived notions, provides significant insights for tackling outbreaks of anthrax and other neglected diseases in Sierra Leone and similar countries.

It is worth mentioning that in 2018, a documented report was published regarding four suspected cases of cutaneous anthrax originating from the Kamakwie region of Sierra Leone [13]. In the report, the diagnosis of all cases was conducted using clinical interviews and enzyme-linked immunosorbent assays [13]. However, the application of this assay for diagnosing anthrax has not been widely endorsed due to its limited accuracy in this particular context [44]. This aspect holds particular significance when considering that the cases under investigation occurred in Kamakwie, an area where anthrax was endemic. Consequently, the exclusion of historical anthrax infection in these cases poses a significant challenge, as it may result in misleading outcomes in antibody-based diagnostic procedures. While the diagnosis of anthrax and similar diseases in Sierra Leone and neighboring countries has been impacted by various challenges [11], this paper presents the first report analyzing *B. anthracis* isolates and clinical specimens from suspected cases of cutaneous anthrax using the qPCR method, which has recently become more accessible and feasible in resource-limited countries due to capacity-building initiatives [11].

The prompt and proactive response from the Ministry of Agriculture and the Ministry of Health and Sanitation to the outbreak played a crucial role in effectively mitigating the risk of spreading. As evidenced by previous investigations [46] and the findings in this study, only six cutaneous anthrax cases were confirmed, all within a month after the implementation of intervention measures. The confirmation of human cases was achieved through qPCR, although attempts to culture *B. anthracis* were unsuccessful. This failure can be attributed to the presence of other bacterial species in the samples, as *B. anthracis* can be readily outcompeted [44]. Consequently, the utilization of molecular techniques for the purpose of tracking human cases presented notable difficulties, thereby raising the question of whether the occurrence of these cases was a consequence of secondary infection stemming from the animal outbreak [6] or if they constituted separate outbreaks. The results obtained from the subsequent long-term active surveillance indicated that the confirmed human cases were predominantly clustered within a limited time period after the outbreak, as opposed to being spread out over the entire duration of the surveillance. This finding provides support for the hypothesis that the human cases resulted from the animal outbreak rather than occurring independently. Notably, despite the absence of confirmed cases during post-outbreak surveillance, it is imperative to avoid underestimating the risk of future anthrax outbreaks, given the prolonged viability of *B. anthracis* spores in the environment, which can persist for several decades [2, 9, 22]. When taking into account the circumstances in which the processing and consumption of contaminated meat occurred locally before the outbreak was officially declared, it is crucial to express considerable concern regarding the repeated occurrence of this disease in Sierra Leone.

The BaSL2022 strain represents the initial identification of the *B. anthracis* isolate discovered in Sierra Leone. The analysis of full-chromosome SNPs revealed that BaSL2022 was classified within the A.Br.153 phylogenetic group and exhibited a close relationship with isolates (Fig. 3B, Fig. 4) obtained from three outbreaks related to drum-making and drumming activities [47] in Scotland (Scotland_484, 2006) [20, 47], London (London_493, 2008) [48], and New York (A3802, 2006) [20, 49]. The animal skins and hides used for the drums associated with the Scottland and New York cases were believed to have been imported from Guinea [20] and Cote d'Ivoire [49], respectively. The London cases were investigated in connection with animal hide sourced from various origins, including Gambia [48]. The observation that the genomes of the strains isolated from the outbreaks were largely similar to each other and constituted a distinct phylogenetic clade (A.Br.153) separate from other existing clades in the TEA lineage (Fig. 3B) further supported the inference that these strains, obtained from cases associated with animal-hide drums, likely originated from the same geographical region, most likely West Africa [20]. However, the verification of this hypothesis was pending, as the three sequences, which were obtained at a later time from samples collected in 2010 in West African Senegal and, specifically, Gambia [23], were found to be different from the sequences obtained from the cases in Scotland, London, and New York. In contrast, a distinct clade known as A.Br.148 [20] was formed, as depicted in Fig. 3B. In light of the aforementioned evidence, our findings present strong support for the conclusive element of the hypothesis, suggesting that the anthrax incidents in the United Kingdom and United States during the 2000s were caused by the spillover of *B. anthracis* from West Africa.

Considering the geographical origins of the hides associated with the outbreaks in Scotland (Guinea), New York (Cote d'Ivoire), and London (multiple origins including Gambia), the identification of BaSL2022 in Sierra Leone has revealed the potential for long-term endemicity of A.Br.153 in at least the west coast of West Africa. Furthermore, it is important to note that all the strains identified in West Africa were found to be genetically distinct from other phylogenetic groups worldwide [12, 23]. Therefore, the association of BaSL2022 with other A.Br.153 isolates signifies the first instance of *B. anthracis* lineages being transmitted from this specific geographical area to other continents.

Our study was subject to certain limitations. Firstly, high-throughput sequencing was not conducted during the initial detection of the outbreak. If a comprehensive analysis had been conducted, it is possible that the outbreak could have been detected and addressed more promptly, resulting in a reduced impact on farmers and lower costs associated with outbreak control. Secondly, the utilization of nanopore sequencing played a pivotal role in the identification of this outbreak. However, the efficiency of nanopore sequencing in RNA sequencing, as opposed to DNA sequencing, is a significant factor to consider. This is particularly true when taking into account the drastic manner in which we processed the samples, which ultimately limited the potential application of our procedure in other situations. Lastly, our attempts to isolate a strain of *B. anthracis* from the samples obtained from human cases were unsuccessful. Therefore, our conclusion regarding the human cases being a result of the animal outbreak was solely derived from the temporal distribution of human cases, without the presence of molecular evidence.

## Conclusions

In the current investigation, the causative agent responsible for a widespread outbreak was successfully identified as *B. anthracis* through the utilization of the nanopore sequencing technique. The initial application of qPCR for the diagnosis of anthrax in this nation resulted in the confirmation of six human cutaneous anthrax cases among 49 suspected cases following the animal anthrax outbreak. We successfully isolated and purified the first Sierra Leonean *B. anthracis* strain BaSL2022 from the outbreak. Phylogenetic analysis revealed that this strain is distinct from the currently available West African lineages and belongs to the A.Br.153 clade. This clade exclusively includes isolates obtained from animal-hide-associated cases in the United Kingdom and United States between 2006 and 2008. This finding provides strong evidence to support the conclusive aspect of the hypothesis that the cases in the United Kingdom and United States were a result of the spillover of *B. anthracis* from West Africa.

In the present circumstances, the lack of awareness about anthrax greatly affected the initial diagnosis and delayed the timely control of the outbreak. Therefore, we propose that governmental bodies and scientific societies take the initiative to launch an intensified educational campaign aimed at raising awareness about anthrax, along with other neglected zoonotic diseases, among farmers, veterinarians, and disease surveillance personnel in Sierra Leone and other similar nations. Furthermore, given the extensive dissemination of contaminated animal meat throughout this outbreak, it is highly advisable to establish a comprehensive and ongoing surveillance system to effectively monitor the potential reoccurrence of anthrax outbreaks as the longevity of *B. anthracis* spores in the environment can span several decades.

### Supplementary Information

**Additional file 1: Table S1: **Annotation of reads obtained from the rapid identification.

## Data Availability

The sequencing data were submitted to the NCBI Sequence Read Archive under the BioProject number PRJNA875505. All the other data yielded in this study are shown in the paper as well as the supplementary materials.

## Abbreviations

cgMLST: Core genome multilocus sequence typing
Ct: Cycle threshold
MLVA: Multilocus variable-number-of-tandem-repeat analysis
qPCR: Quantitative polymerase chain reaction
SNP: Single nucleotide polymorphism
TEA: TransEuroAsian
