# Supplementary material for: Molecular characterization of an outbreak-involved Bacillus anthracis strain confirms the spillover of anthrax from West Africa
Source: Infect Dis Poverty. 2024 Jan 15;13:6. doi: 10.1186/s40249-023-01172-2 (PMC10788998; doi:10.1186/s40249-023-01172-2)
Supplement: Supplementary file 1 — Additional file 1: Table S1: Annotation of reads obtained from the rapid identification. [file 40249_2023_1172_MOESM1_ESM.docx]

**Table S1: Annotation of reads obtained from the rapid identification**

| **Name** | **Tax ID** | **Tax Rank** | **Genome size** | **No. of reads** | **No. of unique reads** | **Abundance** |
| --- | --- | --- | --- | --- | --- | --- |
| Synthetic construct | 32,630 | Species | 26,537,524 | 51 | 49 | 0 |
| *Homo sapiens* | 9606 | Species | 3.24E+09 | 38 | 23 | 0 |
| *Bacillus anthracis* | 1392 | Species | 17,446,789 | 29 | 10 | 0 |
| *Bacillus cereus* | 1396 | Species | 9,676,532 | 18 | 1 | 0 |
| *Vibrio natriegens* | 691 | Species | 10,692,018 | 1 | 0 | 0 |
| *Bacillus thuringiensis* | 1428 | Species | 10,269,592 | 12 | 0 | 0 |
| *Vibrio* sp. EJY3 | 1,116,375 | Species | 5,452,646 | 1 | 0 | 0 |
